# ﻿Morphophylogenetic evidence reveals four new fungal species within Tetraplosphaeriaceae (Pleosporales, Ascomycota) from tropical and subtropical forest in China

**DOI:** 10.3897/mycokeys.100.113141

**Published:** 2023-12-06

**Authors:** Xia Tang, Rajesh Jeewon, Yong-Zhong Lu, Abdulwahed Fahad Alrefaei, Ruvishika S. Jayawardena, Rong-Ju Xu, Jian Ma, Xue-Mei Chen, Ji-Chuan Kang

**Affiliations:** 1 Engineering and Research Center for Southwest Biopharmaceutical Resource of National Education Ministry of China, Guizhou University, Guiyang, 550025, Guizhou Province, China; 2 Center of Excellence in Fungal Research, Mae Fah Luang University, Chiang Rai, 57100, Thailand; 3 School of Science, Mae Fah Luang University, Chiang Rai, 57100, Thailand; 4 Department of Health Sciences, Faculty of Medicine and Health Sciences, University of Mauritius, Reduit, Mauritius; 5 Department of Zoology, College of Science, King Saud University, P.O. Box 2455, Riyadh 11451, Saudi Arabia; 6 School of Food and Pharmaceutical Engineering, Guizhou Institute of Technology, Guiyang, Guizhou Province550003, China; 7 Center for Yunnan Plateau Biological Resources Protection and Utilization, College of Biological Resource and Food Engineering, Qujing Normal University, Qujing 655011, China

**Keywords:** Anamorphic fungi, checklist, Dothideomycetes, ribosomal genes, species diversity, taxonomy

## Abstract

Tetraplosphaeriaceae (Pleosporales, Ascomycota) is a family with many saprobes recorded from various hosts, especially bamboo and grasses. During a taxonomic investigation of microfungi in tropical and subtropical forest regions of Guizhou, Hainan and Yunnan provinces, China, several plant samples were collected and examined for fungi. Four newly discovered species are described based on morphology and evolutionary relationships with their allies inferred from phylogenetic analyses derived from a combined dataset of LSU, ITS, SSU, and *tub2* DNA sequence data. Detailed illustrations, descriptions and taxonomic notes are provided for each species. The four new species of Tetraplosphaeriaceae reported herein are *Polyplosphaeriaguizhouensis*, *Polyplosphaeriahainanensis*, *Pseudotetraploayunnanensis*, and *Tetraploahainanensis*. A checklist of Tetraplosphaeriaceae species with available details on their ecology is also provided.

## ﻿Introduction

The Southwestern part of China is characterised by a tropical to subtropical climate and several provinces are well known for their high diversity of plants as well as fungi (Feng and Yang 2018; Hyde et al. 2020b; Bao et al. 2021; Yang et al. 2023). Yunnan province, for example, is considered a hotspot for species diversity. Over the last few decades, there has been a number of studies that have reported novel fungal species from this region (Jeewon et al. 2003; Luo et al. 2017, 2018; Huang et al. 2018; Su et al. 2018; Yang et al. 2019; Hyde et al. 2020b; Bao et al. 2021; Mortimer et al. 2021). So far more than 6000 fungal species described alone from Yunnan province (Feng and Yang 2018). Guizhou, as a prominent example of China’s karst landform (also referred to as a ‘karst province’), is also characterised by a geomorphological diversity that can be directly related to species diversity. It boasts a distinctive geographical environment and a special climate that fosters the growth of numerous rare, endangered, and indigenous plant, animal, and fungal species. Over the past few decades, extensive research has focused on fungi, encompassing both macro and microfungi, leading to the identification and documentation of roughly over 2,500 fungal species in Guizhou province (Zhou et al. 2018, 2020a, 2020b, 2022; Dissanayake et al. 2020; Chen et al. 2021; Yang et al. 2023). Hainan Province, the largest island in the Indo-Burma biodiversity hotspot, contains extensive and well-preserved tropical forests (Huang et al. 2023). Recent studies have indicated the presence of diverse fungal species in Hainan, with roughly over 1000 fungal species. Most of these fungi are macrofungi (Zhang et al. 1994; Li et al. 2010; Hapuarachchi et al. 2018; Huang et al. 2023). With our current fungal biodiversity estimates and given that mycologists anticipate many more species remain to be discovered especially in explored habitats, this research study has been undertaken to investigate microfungi in this region, and potentially discover new fungal species.

Tetraplosphaeriaceae was introduced by Tanaka et al. (2009) to accommodate the massarina-like species that produced tetraploa-like anamorphs in culture with *Tetraplosphaeria* as its type genus. Tetraplosphaeriaceae is known to be widely distributed on various hosts, with most species reported from bamboo or grasses (Poaceae) (Tanaka et al. 2009; Ariyawansa et al. 2015; Li et al. 2016), while *Tetraploa* species occur on diverse hosts (Hyde et al. 2013). Tetraplosphaeriaceae was first described to accommodate five genera: *Polyplosphaeria* Kaz. Tanaka & K. Hiray, *Pseudotetraploa* Kaz. Tanaka & K. Hiray, *Quadricrura* Kaz. Tanaka & K. Hiray, *Tetraplosphaeria* Kaz. Tanaka & K. Hiray, and *Triplosphaeria* Kaz. Tanaka & K. Hiray (Tanaka et al. 2009). Hyde et al. (2013) treated the type genus, *Tetraplosphaeria* as a synonym of *Tetraploa* according to nomenclatural priority. Ariyawansa et al. (2015) accepted *Shrungabeeja* in Tetraplosphaeriaceae based on morphological and molecular evidence from *S.longiappendiculata*. Later, *Ernakulamia* was accommodated in Tetraplosphaeriaceae based on morphology and phylogenetic analyses of combined ITS, LSU and *tub2* sequence data (Delgado et al. 2017; Hyde et al. 2020a). Pem et al. (2019) transferred *Byssolophis* from the genera incertae sedis to Tetraplosphaeriaceae based on its massarina-like morphology and phylogenetic analyses based on combined LSU, SSU, *tef1-α*, and *rpb2* sequence data. Hongsanan et al. (2020) provided a taxonomic update on families of Dothideomycetes and eight genera were accepted in Tetraplosphaeriaceae. Recently, Li et al. (2021) discovered a freshwater fungus that had a close phylogenetic affinity with *Ernakulamia* and *Shrungabeeja* in Tetraplosphaeriaceae and accommodated it in a new genus *Aquatisphaeria* based on morphology and phylogeny. To date, Tetraplosphaeriaceae consists of nine genera (Hongsanan et al. 2020; Li et al. 2021; Wijayawardene et al. 2022).

Most members of Tetraplosphaeriaceae contain anamorphic species (Wijayawardene et al. 2022). However, *Pseudotetraploa*, *Tetraploa*, and *Triplosphaeria* exhibit both teleomorphs and anamorphs (Wijayawardene et al. 2022), while *Byssolophis* is only known in its teleomorphic morph (Tanaka et al. 2009; Ariyawansa et al. 2015; Pem et al. 2019; Hongsanan et al. 2020; Li et al. 2021; Jayawardena et al. 2023). Tetraplosphaeriaceae is characterized by massarina-like teleomorph morphs but can be distinguished from other families by its immersed to superficial, glabrous or brown hyphae at sides of ascomata with flattened bases and cylindrical to clavate, short pedicellate 8-spored asci which are narrowly fusiform to broadly cylindrical, septate, hyaline to pale brown ascospores, usually with a complete sheath or appendage-like sheath (Tanaka et al. 2009; Hyde et al. 2013). The anamorphs are tetraploa-like hyphomycetes having micronematous to macronematous, erect, unbranched, septate, with presence or absence of conidiophores, monoblastic, terminal conidiogenous cells sometimes indistinguishable from creeping hyphae, solitary, cylindrical to obpyriform, comprising 3–8 columns or internal hyphal structure conidia, mostly verrucose at the conidial base and with 2–8-setose appendages (Tanaka et al. 2009; Hyde et al. 2013; Hongsanan et al. 2020).

In this study, the aim is to characterize anamorphic fungal species collected from the southern part of China. The objectives are to 1) to describe novel species collected from Guizhou, Hainan, and Yunnan provinces in China, based on morphological examination of fresh specimens; 2) to document morphological differences and similarities with extant species; 3) to establish four new species within the family Tetraplosphaeriaceae with support from results generated from phylogenetic analyses of LSU, ITS, SSU, and *tub2* DNA sequence data; 4) to provide a worldwide checklist of Tetraplosphaeriaceae species with available details on their ecology.

This study will undoubtedly increase our understanding of fungal diversity in China.

## ﻿Materials and methods

### ﻿Sample collection, isolation and morphological studies

Fresh samples of unidentified decaying wood and decaying bamboo were collected in Guizhou (Xingyi city, Xianheping National Forest Park), Hainan (Wuzhishan city, Wuzhishan National Nature Reserve), and Yunnan (Puer city, Ailao mountains) provinces respectively. During the collection period, the environmental conditions at the different regions were as follows: Guizhou-average temperature of 26 °C, subtropical climate, humid environment during autumn; Hainan-average temperature of 29 °C, tropical climate, humid environment during autumn; Yunnan-average temperature of 22 °C, subtropical climate, humid environment during spring. The samples were placed in Ziplock bags, labelled with a marker pen, and observed using the stereomicroscope (Motic SMZ-171). The procedure for specimen collection, observation and isolation follows that of Senanayake et al. (2020) and Tang et al. (2022). The morphological measurements were performed by the Tarosoft (R) Image Frame Work tool (IFW 0.97 version), and photoplates were created using the Adobe Photoshop 2019 program (Adobe Systems, USA).

After morphological examination, the specimens were deposited at the herbaria of Kunming Institute of Botany, Chinese Academy of Sciences (**HKAS**), Kunming, China, and the Guizhou Academy of Agriculture Sciences (**GZAAS**), Guiyang, China, respectively. The ex-type cultures were deposited at the Guizhou Culture Collection (**GZCC**) in China and the Kunming Institute of Botany Culture Collection (**KUNCC**). Faces of Fungi and Index Fungorum numbers are provided as in Jayasiri et al. (2015) and Index Fungorum (2023). Species recognition and justifications for new species establishment were done based on the guide-lines provided by Jeewon and Hyde (2016), Chethana et al. (2021) and Pem et al. (2021).

### ﻿DNA extraction, PCR amplification and sequencing

Fresh mycelium was scraped from the living culture and transferred to 1.5 mL microcentrifuge tubes and kept in a refrigerator at -20 °C. Total genomic DNA was extracted using the DNA extraction kits (Sangon Biotech (Shanghai) Co. Ltd., China). DNA template amplifications were performed by Polymerase Chain Reaction (PCR) using primer pairs, ITS5/ITS4 for ITS (White et al. 1990), NS1/NS4 for SSU (White et al. 1990), LR0R/LR5 for LSU (Vilgalys and Hester 1990, Cubeta et al. 1991), and BT1/BT2b for *tub2* (Glass and Donaldson 1995). For other details pertaining to DNA extraction, PCR amplifications, sequencing, and phylogenetic analyses, see Tang et al. (2022). The polymerase chain reaction was carried out in a volume of 50 μL, and the reagents that were used were as follows: DNA template (2 μL), forward primers (2 μL), reverse primers (2 μL), 2 ×Taq PCR Master Mix (25 μL) and 19 μL of ddH_2_O (double-distilled water). PCR profiles are as follows: 35 cycles, and the annealing temperatures for each gene are 52 °C for 1 minute and extension at 72 °C for 90 seconds in LSU, ITS and SSU; and 55 °C for 50 s and elongation at 72 °C for 1 minute for tub2. Verification of PCR products was done on 1% agarose gels before being sent to China’s Sangon Biotech (Shanghai) Co., Ltd. for sequencing.

### ﻿Phylogenetic analyses

The forward and reverse primers of the newly generated sequence were assembled by the Contig Ex-press v3.0.0 application, and the most similar taxa were found by BLASTn (https://blast.ncbi.nlm.nih.gov/Blast.cgi) in NCBI. A combination of different DNA sequence data (LSU, ITS, SSU, and *tub2*), which are close hits and similar to other Tetraplosphaeriaceae species in GenBank (Table 1), were downloaded to be further analysed along with our new taxa. Each sequence data was aligned by the online version of MAFFT v. 7 (https://mafft.cbrc.jp/alignment/server/index.html) through the “auto” option (Katoh et al. 2017). Multiple genes were assembled by SequenceMatrix (Vaidya et al. 2011). The aligned sequence was trimmed by trimAl v 1.2 with the ‘gappyout’ option (Capella-Gutiérrez et al. 2009). The phylogenetic analyses in this study were based on the maximum likelihood (ML), and Bayesian inference (BI) by using a combined sequence dataset of LSU, ITS, SSU, and *tub2*.

**Table 1.** Taxa used in this study and their GenBank accession numbers for LSU, ITS, SSU and *tub2* sequence data.

Taxa name	Strain Numbers	GenBank Accession Numbers			
		LSU	SSU	ITS	*tub2*
*Amniculicolaimmersa*	CBS 123083T	NG_056964	NG_062796	–	–
*A.parva*	CBS 123092T	NG_056970	NG_016504	–	–
*Aquatisphaeriathailandica*	MFLUCC 21–0025T	MW890763	MW890967	MW890969	–
*Aq.Thailandica*	DLUCC B151	MW890764	MW890968	–	–
*Byssolophissphaerioides*	IFRDCC 2053	GU301805	GU296140	–	–
*Ernakulamiacochinensis*	MFLUCC 18–1237	MN913716	MT864326	MT627670	–
*E.krabiensis*	MFLUCC 18–0237T	MK347990	MK347880	MK347773	–
*E.tanakae*	NFCCI 4615T	MN937211	–	MN937229	MN938312
*E.xishuangbannaensis*	KUMCC 17–0187T	MH260314	MH260354	MH275080	–
*Polyplosphaeriafusca*	KT 2124	AB524607	AB524466	AB524791	AB524853
*Po.Fusca*	KT 1616T	AB524604	AB524463	AB524789	AB524851
***Po.guizhouensis***	**GZCC 23–0598T**	**OR438888**	–	**OR427327**	**OR449118**
***Po.Hainanensis***	**GZCC 23–0599T**	**OR438889**	**OR438285**	**OR427323**	**OR449115**
***Po.Hainanensis***	**GZCC 23–0600**	**OR438890**	–	**OR427324**	–
*Po.Thailandica*	MFLUCC 15–0840T	KU248767	–	KU248766	–
*Po.nabanheensis*	KUMCC 16–0151T	MH260312	MH260352	MH275078	MH412745
*Po.pandanicola*	MFLUCC 17–2266T	MH260313	MH260353	MH275079	–
*Pseudotetraploabambusicola*	CGMCC 3.20939T	ON332933	ON332923	ON332915	–
*Ps.bambusicola*	UESTCC 22.0005	ON332934	ON332924	ON332916	–
*Ps.curviappendiculata*	JCM 12852T	AB524608	AB524467	AB524792	AB524854
*Ps.Javanica*	JCM 12854	AB524611	AB524470	AB524795	AB524857
*Ps.Longissima*	JCM 12853T	AB524612	AB524471	AB524796	AB524858
*Ps.rajmachiensis*	NFCCI 4618T	MN937204	–	MN937222	–
***Ps.Yunnanensis***	**KUNCC 10464**T	**OR438891**	–	**OR449073**	–
*Quadricrurabicornis*	CBS 125427T	AB524613	AB524472	AB524797	AB524859
*Q.meridionalis*	CBS 125684T	AB524614	AB524473	AB524798	AB524860
*Q.septentrionalis*	CBS 125429	AB524615	AB524474	AB524799	AB524861
*Shrungabeejaaqutica*	MFLUCC 18–0664T	MT627663	–	MT627722	–
*S.longiappendiculata*	BCC 76463T	KT376472	KT376471	KT376474	–
*S.longiappendiculata*	BCC 76464	KT376473	–	KT376475	–
*S.fluviatilis*	GZCC 20–0505T	–	–	–	–
*S.fluviatilis*	GZCC 19–0511	MW133853	MW134631	–	–
*S.vadirajensis*	MFLUCC 17–2362	MN913685	–	MT627681	–
*Tetraploaaquatica*	MFLU 19–0996	MT530453	MT530454	MT530449	–
*T.aquatica*	MFLUCC 19–0995T	MT530452	–	MT530448	–
*T.aristata*	CBS 996.70	AB524627	AB524486	AB524805	AB524867
*T.bambusae*	KUMCC 21–0844T	ON077067	ON077073	ON077078	ON075065
*T.dwibahubeeja*	NFCCI 4621T	MN937207	–	MN937225	MN938308
*T.dwibahubeeja*	NFCCI 4623	MN937208	–	MN937226	MN938309
*T.endophytica*	CBS 147114T	MW659165	–	KT270279	–
***T.hainanensis***	**GZCC 23–0601T**	**OR438892**	**OR438286**	**OR427325**	**OR449116**
***T.hainanensis***	**GZCC 23–0602**	**OR438893**	–	**OR427326**	**OR449117**
*T.juncicola*	CBS 149046	ON603800	–	ON603780	–
*T.nagasakiensis*	KUMCC 18–0109	MK079891	MK079888	MK079890	–
*T.nagasakiensis*	KT 1682T	AB524630	AB524489	AB524806	AB524868
*T.pseudoaristata*	NFCCI 4624T	MN937214	–	MN937232	MN938315
*T.pseudoaristata*	NFCCI 4625	MN937212	–	MN937230	MN938313
*T.puzheheiensis*	MFLUCC 20–0151T	MT627655	–	MT627744	–
*T.sasicola*	KT 563T	AB524631	AB524490	AB524807	AB524869
*T.thailandica*	MFLUCC 21–0030T	MZ412530	MZ413274	MZ412518	–
*T.thrayabahubeeja*	NFCCI 4627T	MN937217	–	MN937235	MN938318
*T.thrayabahubeeja*	NFCCI 4628	MN937215	–	MN937233	MN938316
*T.yunnanensis*	MFLUCC 19–0319T	MN913735	MT864341	MT627743	–
*T.yakushimensis*	KT 1906T	AB524632	AB524491	AB524808	AB524870
*T.cylindrica*	KUMCC 20–0205T	MT893204	MT893203	MT893205	MT899417
*T.cylindrica*	ZHKUCC 22–0087	ON555688	ON555690	ON555689	ON564477
*T.dashaoensis*	KUMCC 21–0010T	OL473555	OL473556	OL473549	OL505601
*T.obpyriformis*	KUMCC 21–0011T	OL473554	OL473557	OL473558	OL505600
*Tetraploa* sp.1	KT 1684	AB524628	AB524487	–	–
*Tetraploa* sp.2	KT 2578	AB524629	AB524488	–	–
*T.tetraploa*	CY112	–	–	HQ607964	–
*Triplosphaeriaacuta*	KT 1170T	AB524633	AB524492	AB524809	AB524871
*Tr.Cylindrica*	NBRC 106247	AB524636	AB524495	AB524811	AB524873
*Tr.cylindrica*	KT 1800	AB524635	AB524494	AB524810	AB524872
*Tr.maxima*	KT 870T	AB524637	AB524496	AB524812	AB524874
*Tr.yezoensis*	KT 1715T	AB524638	AB524497	AB524813	AB524875
*Tr.yezoensis*	KT 1732	AB524639	AB524498	AB524814	AB524876
*Triplosphaeria* sp.	HHUF 27481	AB524640	AB524499	AB524815	AB524877
*Triplosphaeria* sp.	KT 2546	AB524641	AB524500	AB524816	AB524878

Notes: Ex-type strains are indicated by “T” at the end of the strain number, and newly generated sequences are in bold. Abbreviations: **BCC**: Biotec Culture Collection, Bangkok, Thailand; **CBS**: Westerdijk Fungal Biodiversity Institute, Utrecht, the Netherlands; **CGMCC**: China General Microbiological Culture Collection Centre, Beijing, China; **DLUCC**: Dali University Culture Collection, Yunnan, China; **GZCC**: Guizhou Culture Collection, Guizhou, China; **HHUF**: Herbaria of Hirosaki University; **IFRDCC**: Culture Collection, International Fungal Research and Development Centre, Chinese Academy of Forestry, Kunming, China; **JCM**: the Japan Collection of Microorganisms, Japan; **KUNCC**: Kunming Institute of Botany Culture Collection; **KT**: Kazuaki Tanaka; **MFLUCC**: Mae Fah Luang University Culture Collection, Chiang Rai, Thailand; **MFLU**: Mae Fah Luang University Herbarium Collection; **NBRC**: Nite Biological Resource Center, Department of Biotechnology, National Institute of Technology and Evaluation, Japan; **NFCCI**: National Fungal Culture Collection of India NFCCI-A National Facility; **UESTCC**: University of Electronic Science and Technology Culture Collection, Chengdu, China; **ZHKUCC**: Zhongkai University of Agriculture and Engineering Culture Collection, Guangzhou, China. ***A.*** = *Amniculicola*. ***Aq.*** = *Aquatisphaeria*. ***E.*** = *Ernakulamia.****Po.*** = *Polyplosphaeria*. ***Ps.*** = *Pseudotetraploa*. ***Q.*** = *Quadricrura*. ***S.*** = *Shrungabeeja*. ***T.*** = *Tetraploa*. ***Tr.*** = *Triplosphaeria*.

Analyses under different criteria such as maximum likelihood (ML) and Bayesian inference (BI) were processed in the CIPRES web portal (Miller et al. 2010) by using the “RAxML-HPC v.8 on XSEDE” tool, and the tool “MrBayes on XSEDE”, respectively (Huelsenbeck and Ronquist 2001; Swofford 2002; Stamatakis et al. 2008; Ronquist et al. 2012).

For BI, MrModeltest v2 was used for the selection of the best-fit model for each gene region. The Markov Chain Monte Carlo (MCMC) algorithm was launched with four chains running concurrently from a random tree topology. The burn-in factor was set at 25%, and the sampling interval for trees was set to every 1000^th^ generation. The Posterior Probabilities (PP) for the remaining trees were computed. Adobe Illustrator and FigTree were used to view trees. Bootstrap support and Bayesian posterior probabilities above 70 and 0.9 were considered as high support respectively.

## ﻿Results

### ﻿Phylogenetic analyses

**Figure 1.**
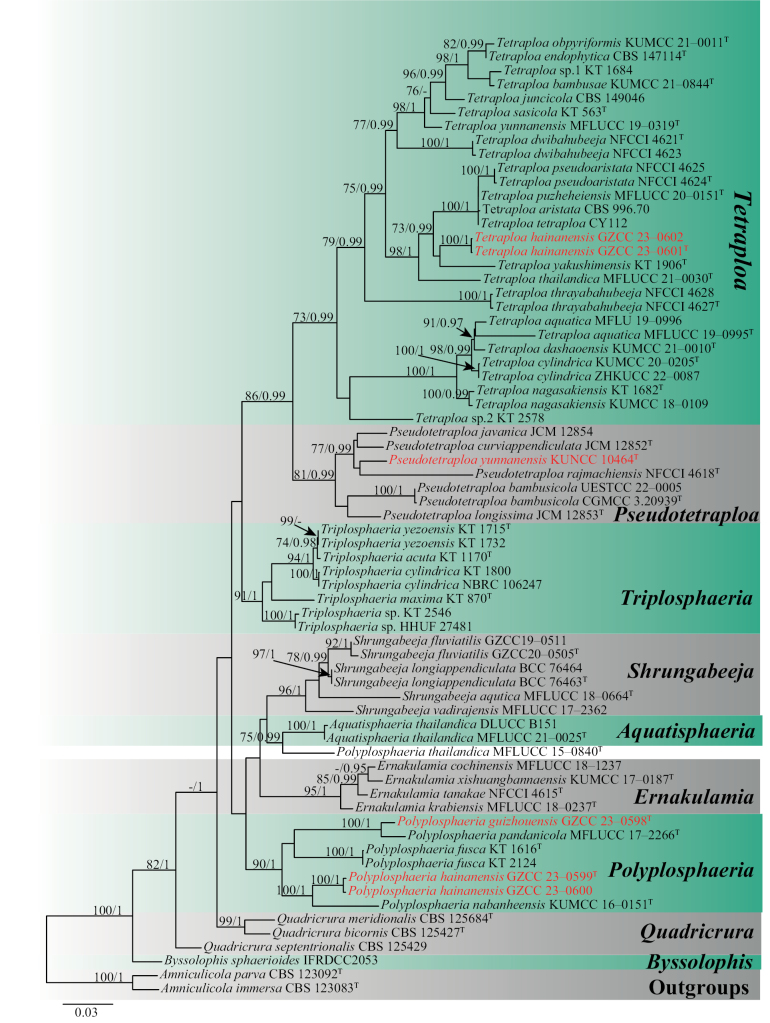
Phylogenetic construction of Tetraplosphaeriaceae using RAxML-based maximum likelihood analysis of a combined LSU, ITS, SSU, and *tub2* DNA sequence dataset. Bootstrap support values for maximum likelihood (ML) equal to or greater than 70% and Bayesian posterior probabilities (PP) equal to or greater than 0.95 PP are shown above the nodes. The tree is rooted with *Amniculicolaimmersa* (CBS 123083) and *A.parva* (CBS 123092). Newly generated strains are in red, and the type strains are indicated using “T” in superscript.

For the phylogenetic analyses, a combined DNA sequence data of 68 taxa on LSU, ITS, SSU, and *tub2* was used and analysed under the ML and PP criteria. The data matrix comprised 2995 total characters, including gaps (LSU: 1–848 bp, ITS: 849–1372 bp, SSU: 1373–2363 bp, *tub2*: 2364–2995 bp). Phylogenetic reconstructions with broadly comparable topologies were recovered from the combined dataset of ML and PP analyses. The top-scoring RAxML tree is shown in Fig. 1, with a final ML optimization likelihood value of -17569.286960 (ln). In the RAxML analysis, the GTRGAMMA+I-Invar model was used and the results showed 969 unique alignment patterns and 25.50% of indeterminate characters. Base frequency estimates were as follows: A = 0.243260, C = 0.247998, G = 0.277213, T = 0.231530; substitution rates were as follows: AC = 3.027135, AG = 4.828263, AT = 2.159193, CG = 1.385950, CT = 10.517436, GT = 1.000000; gamma distribution shape parameter alpha = 0.166037; and tree-length has been as follows: 1.837225. The best-fit models for the BPP analysis were GTR+I+G for LSU, ITS, and *tub2* gene regions; HKY+I+G for the SSU gene region. With a final average standard deviation of split frequencies of 0.009909, Bayesian posterior probabilities from MCMC were analysed. The new taxa analysed herein all belong to the Tetraplosphaeriaceae clade based on the results of the combined LSU, ITS, SSU, and *tub2* sequence data analysis.

## ﻿Taxonomy

### ﻿ Tetraplosphaeriaceae

Taxon classificationFungiPleosporalesTetraplosphaeriaceae

Kaz. Tanaka & K. Hiray, Studies in Mycology 64: 177 (2009)

5A855477-A108-5E95-904B-8552F57D7120

515253

Facesoffungi Number: FoF06665

#### Type genus.

*Tetraploa* Berk. & Broome, Ann. Mag. Nat. Hist. 5: 459, t. 11:6 (1850).

#### Description.

***Teleomorph*** see Tanaka et al. (2009). ***Anamorph*** Conidiophores absent. Conidiogenous cells monoblastic. Conidia composed of 3–8 columns or internal hyphal structure, brown, mostly verrucose at the base, with more than 3–8 setose appendages (Tanaka et al. 2009).

#### Notes.

Tetraplosphaeriaceae was described by Tanaka et al. (2009) to accommodate the species which has massarina-like teleomorphic morph and tetraploa-like anamorphs based on a combined SSU and LSU DNA sequence data and established five genera. To date, the members of Tetraplosphaeriaceae are mainly distributed on Poaceae and unidentified decayed wood as saprobes and pathogens from aquatic and terrestrial habitats (Tanaka et al. 2009; Hyde et al. 2013; Hongsanan et al. 2020; Yu et al. 2022; Li et al. 2021). It now contains nine genera and 69 species (Tanaka et al. 2009; Pem et al. 2019; Hongsanan et al. 2020; Li et al. 2021; Liao et al. 2022).

### ﻿ Polyplosphaeria

Taxon classificationFungiPleosporalesTetraplosphaeriaceae

Kaz. Tanaka & K. Hirayama, Studies in Mycology 64: 192 (2009)

A7868195-79B5-543F-80D2-4780ADC45C31

515256

Facesoffungi Number: FoF06668

#### Type species.

*Polyplosphaeriafusca* Kaz. Tanaka & K. Hirayama, Studies in Mycology 64: 193 (2009).

#### Description.

***Teleomorph*** see Tanaka et al. (2009). ***Anamorph*** Conidiophores absent. Conidiogenous cells monoblastic. Conidia globose to subglobose, with thin peel-like outer wall of conidia, composed of numerous internal hyphae at the inside, brown, almost smooth, verrucose at the base. Appendages with 3 to 8 setose appendages, brown, straight (Tanaka et al. 2009).

#### Notes.

Tanaka et al. (2009) established *Polyplosphaeria* and typified with *Po.fusca* based on a combined SSU and LSU DNA sequence data. All the members of *Polyplosphaeria* were reported as saprobes from various plant hosts, such as *Pleioblastuschino*, *Phyllostachysbambusoides* and Pandanaceae (Tanaka et al. 2009; Li et al. 2016; Tibpromma et al. 2018). *Polyplosphaeria* is distributed in Japan, China and Thailand in terrestrial habitats (Tanaka et al. 2009; Li et al. 2016; Tibpromma et al. 2018). Dong et al. (2020) transferred *Po.xishuangbannaensis* into *Ernakulamia* based on phylogenetic analyses and differences in morphology. In this study, two new *Polyplosphaeria* species are introduced from unidentified decaying wood from China. The genus contains six species *viz. Polyplosphaeriaguizhouensis*, *Po.hainanensis*, *Po.fusca*, *Po.nabanheensis*, *Po.pandanicola* and *Po.thailandica* (Tanaka et al 2009; Li et al. 2016; Tibpromma et al. 2018; This study; Table 2).

**Table 2.** Tetraplosphaeriaceae species and their country, life cycle, habitat, host and reference.

Species name	Country	Life cycle	Habitat	Host	Reference
*Aquatisphaeriathailandica*	Thailand	saprobic	freshwater	decaying wood	Li et al. (2021)
*Byssolophisbyssiseda*	France	saprobic	terrestrial	branch of *Carpinus*, decaying wood	Zhang et al. (2012)
*B.sphaerioides*	Finland, UK	saprobic	terrestrial	decaying stemp of *Rubus*, decaying wood birch	Berkeley and Broome (1854); Karsten (1870)
*Ernakulamiacochinensis*	Argentina, Cuba, India, Japan, Malaysia, Mexico, Panama, Thailand	saprobic	freshwater, terrestrial	*Astrocaryumstandleyanum*, *Benthamidiajaponica*, dead leaves, dead spathes of *Cocosnucifera*, decomposing leaves of *Satakentialiukivensis*, *Freycinetiamulti*, palm tree, *Ilex* sp., *Ocotealeucoxylon*, *Pandanustectorius*, *P.monticola*, submerged wood, *Syagrusromanzoffiana*, *Stewartiamonadelpha*, *Vitex* sp.	Ellis (1976); Holubová-Jechová and Mercado (1986); Holubová-Jechová (1989); Mercado et al. (1997, 2005); Taylor and Hyde (2003); Delgado and Mena (2004); Capdet and Romero (2010); Whitton et al. (2012); Delgado et al. (2017); Dong et al. (2020); Farr and Rossma (2023)
*E.krabiensis*	Thailand	saprobic	terrestrial	*Acacia* sp.	Jayasiri et al. (2019)
*E.tanakae*	India	saprobic	terrestrial	dead spathes of *Cocosnucifera*	Hyde et al. (2020a)
*E.xishuangbannaensis*	China	saprobic	terrestrial	dead leaves of *Pandanus* sp.	Tibpromma et al. (2018); Dong et al. (2020)
***Polyplosphaeriaguizhouensis***	**China**	**saprobic**	**terrestrial**	**unidentified decaying wood**	**This study**
***Po.Hainanensis***	**China**	**saprobic**	**terrestrial**	**unidentified decaying wood**	**This study**
*Po.Fusca*	Japan	saprobic	terrestrial	culms of *Chimonobambusamarmorea*, culms of *Phyllostachysbambusoides*, culms of *Pleioblastuschino*, culms of *Sasakurilensis*	Tanaka et al. (2009)
*Po.thailandica*	Thailand	saprobic	terrestrial	decaying bamboo	Li et al. (2016)
*Po.nabanheensis*	China	saprobic	terrestrial	decaying leaves of *Pandanus* sp.	Tibpromma et al. (2018)
*Po.pandanicola*	China	saprobic	terrestrial	decaying leaves of *Pandanus* sp.	Tibpromma et al. (2018)
*Pseudotetraploabambusicola*	China	saprobic	terrestrial	dead branches of Bamboo	Yu et al. (2022)
*Ps.curviappendiculata*	Japan	saprobic	terrestrial	culms of *Sasakurilensis*	Tanaka et al. (2009)
***Ps.yunnanensis***	**China**	**saprobic**	**freshwater**	**bamboo**	**This study**
*Ps.Javanica*	Indonesia, Japan	saprobic	terrestrial	culms of decaying *Bambusaglaucescens*, culms of *Phyllostachysbambusoides*, culms of *Pleioblastuschino*, culms of *Sas*a sp., dead bark of broad-leaved tree, dead stems of an unidentified herbaceous plant	Hatakeyama et al. (2005); Tanaka et al. (2009); Rifai et al. (2014)
*Ps.longissima*	Japan	saprobic	terrestrial	culms of *Pleioblastuschino*	Tanaka et al. (2009)
*Ps.rajmachiensis*	India	saprobic	terrestrial	decaying bamboo culms, *Dendrocalamusstocksii* (Poaceae)	Hyde et al. (2020a)
*Quadricrurabicornis*	Japan	saprobic	terrestrial	culms of *Sasakurilensis*, leaf litter of a conifer	Tanaka et al. (2009)
*Q.meridionalis*	Japan	saprobic	terrestrial	bamboo	Tanaka et al. (2009)
*Q.septentrionalis*	Japan	saprobic	terrestrial	culms of *Sasakurilensis*	Tanaka et al. (2009)
*Shrungabeejaaquatica*	Thailand	saprobic	freshwater	submerged wood	Dong et al. (2020)
*S.longiappendiculata*	Thailand	saprobic	terrestrial	dead culm of *Bambusa* sp. (Poaceae)	Ariyawansa et al. (2015)
*S.vadirajensis*	Brazil, China, India	saprobic	terrestrial	dead branches of unidentified plant	Rao and Reddy (1981); Zhang et al. (2009)
*S.begoniae*	China	saprobic	terrestrial	dead branches of *Begoniasemperflorens*	Zhang et al. (2009)
*S.melicopes*	China	saprobic	terrestrial	dead branches of *Melicopetriphylla*	Zhang et al. (2009)
*S.piepenbringiana*	Panama	saprobic	terrestrial	dead Poaceae	Kirschner et al. (2017)
*S.fluviatilis*	China	saprobic	freshwater	submerged decaying twig	Yang et al. (2023)
*Tetraploaabortiva*	Argentina	saprobic	freshwater	N/A	Arambarri et al. (1987)
*T.aquatica*	China	saprobic	freshwater	submerged decaying wood	Li et al. (2020)
*T.aristata*	Africa, Barbados, Bolivia, China, Cuba, Denmark, Eire, Europe, Fiji, Germany, Ghana, Hong Kong (China), India, Italy, Japan, Jamaica, Malaysia, Nepal, New Caledonia, Pakistan, Papua New Guinea (New Britain), Philippines, Puerto Rico, Sierra Leone, Thailand, The Dominican Republic, The Netherlands, Uganda, Venezuela, USA(Alabama)	pathogenic (human), saprobic	terrestrial	*Alpiniaformosa*, *Ammophilaarenaria*, *Anadelphialeptocoma*, *Andropogon*, *Angelicasylvestris*, *Avenapralensis*, *Axonopus, Bambusa*, *Carexpaniculata*, *Cladiummariscus*, *Cladiumselloana*, Cocos, *Cortaderia*, *Cymbopogonafronardus*, *Cyperuslongus*, *Dactylis*, *Deschampsia*, *Erianthus*, *Euchlaena*, *Festuca*, *Gyneriumargenteum*, Gynerium, *Heracleumsphondylium*, *Heteropogon*, *Heveabrasiliensis*, *Juncus*, *Musa*, *Phalarisarundinacea*, *Phaseolus*, *Phoenix*, *Phormium, Phragmitescommunis*, *Poapratensis*, *Pteridiumaquilinum*, *Saccharumofficinarum*, *Sorghum*, straw, *Triticum*, unnamed host, wheat stubble, *Zea*	Ellis (1949, 1971); Markham et al. (1990); Tanaka et al. (2009); Senwanna et al. (2021)
*T.bambusae*	China	saprobic	terrestrial	dead twigs of bamboo	Phookamsak et al. (2022)
*T.biformis*	Japan	saprobic	terrestrial	dead bark of broad-leaved tree	Matsushima and Matsushima (1996)
*T.circinata*	India	saprobic	terrestrial	decaying bamboo twig	Pratibha and Bhat (2008)
*T.conata* F	India	N/A	N/A	N/A	Saxena and Sarkar (1986); Gupta (2002)
*T.cylindrica*	China	saprobic	terrestrial	decaying stems of *Saccharumarundinaceum* (Poaceae)	Liao et al. (2022)
*T.dashaoensis*	China	saprobic	terrestrial	dead stem of *Saccharumarundinaceum*	Jayawardena et al. (2023)
*T.divergens*	USA (Mississippi)	saprobic	terrestrial	leaves of *Panicumagrostidiforme*	Tracy and Earle (1895)
*T.dwibahubeeja*	India	saprobic	terrestrial	decaying spathes of *Cocosnucifera*	Hyde et al. (2020a)
*T.ellisii*	Argentina, USA (New Jersey), Zimbabwe	saprobic	terrestrial	*Chloris*, *Dactylis*, *Heveabrasiliensis*, stalks of *Zeamays*	Cooke and Ellis (1879); Ellis (1971); Senwanna et al. (2021)
*T.endophytica*	Germany	endophytic	terrestrial	roots of *Microthlaspiperfoliatum*	Crous et al. (2021)
***T.hainanensis***	**China**	**Saprobic**	**terrestrial**	**unidentified decaying wood**	**This study**
*T.indica* ^F^	India	N/A	N/A	N/A	Saxena and Khare (1991)
*T.josettae* ^F^	France	N/A	N/A	N/A	Nuñez Otaño et al. (2022)
*T.juncicola*	The Netherlands	saprobic	terrestrial	dead culm of *Juncusinflexus* (Juncaceae)	Crous et al. (2022)
*T.muscicola*	Spain	N/A	N/A	fronds of *Aneuramultifida*, *Lophoziaquinquedentata*	González Fragoso (1916)
*T.nagasakiensis*	Japan, China	saprobic	terrestrial	culms of bamboo	Hyde et al. (2013, 2019)
*T.obpyriformis*	China	saprobic	terrestrial	dead grass under *Saccharumarundinaceum* (Gramineae)	Unpublished
*T.opaca*	China	saprobic	terrestrial	dead culms of bamboo, decaying branches of unidentified tree	Zhao et al. (2009)
*T.pseudoaristata*	India	saprobic	terrestrial	decaying spathes of *Cocosnucifera* (Arecacceae)	Hyde et al. (2020a)
*T.puzheheiensis*	China	saprobic	freshwater	submerged wood	Dong et al. (2020)
*T.sasicola*	China, Japan	saprobic	terrestrial	culms of *Sasasenanensis*, dead leaves of *Pennisetumpurpureum* (Poaceae)	Tanaka et al. (2009); Hyde et al. (2020a)
*T.scabra*	USA	N/A	terrestrial	*Scirpus* sp.	Harkness (1885)
*T.scheueri*	UK	saprobic	freshwater, terrestrial	leaves of *Carexacutiformis*, rotten leaves	Scheuer (1991); Hyde et al. (2013)
*T.setifera*	Hungary	saprobic	terrestrial	rotten wood	Révay (1993)
*T.siwalika* ^F^	N/A	N/A	N/A	N/A	Saxena et al. (1987)
*T.taugourdeaui* ^F^	India	N/A	N/A	N/A	Saxena and Sarkar (1986)
*T.thailandica*	Thailand	saprobic	freshwater	Submerged decaying wood	Bao et al. (2021)
*T.thrayabahubeeja*	India	saprobic	terrestrial	decaying spathes of *Cocosnucifera* (Arecacceae)	Hyde et al. (2020a)
*T.yakushimensis*	Japan	saprobic	terrestrial	culms of *Arundodonax*	Tanaka et al. (2009); Hyde et al. (2013)
*T.yunnanensis*	China, Thailand	saprobic	freshwater	submerged wood	Dong et al. (2020)
*Tetraploa* sp. 1	Japan	saprobic	terrestrial	culms of bamboo	Tanaka et al. (2009)
*Tetraploa* sp. 2	Japan	saprobic	terrestrial	culms of Gramineae	Tanaka et al. (2009)
*Triplosphaeriacylindrica*	Japan	saprobic	terrestrial	culms of *Sasakurilensis*	Tanaka et al. (2009)
*Tr.maxima*	Japan	saprobic	terrestrial	culms of *Sasakurilensis*	Tanaka et al. (2009)
*Tr.yezoensis*	Japan	saprobic	terrestrial	culms of *Sasapalmata*	Tanaka et al. (2009)
*Tr.acuta*	Japan	saprobic	freshwater	submerged culms of bamboo	Tanaka et al. (2009)
*Triplosphaeria* sp.	Japan	saprobic	terrestrial	culms of *Sasakurilensis*	Tanaka et al. (2009)

Fossil fungi were indicated “F”; N/A: Not available or cannot find; Newly species indicate in bold.

### ﻿ Polyplosphaeria guizhouensis

Taxon classificationFungiPleosporalesTetraplosphaeriaceae

X. Tang, Jayaward., R. Jeewon & J.C. Kang sp. nov.

BA06DDA4-BFBB-5066-8618-7FE7F84CD3C3

900950

Facesoffungi Number: FoF14571

Fig. 2

#### Etymology.

The specific epithet ‘*guizhouensis*’ refers to the place where the fungus was collected, Guizhou Province, China.

#### Holotype.

GZAAS 23–0600.

#### Description.

***Saprobic*** on unidentified decaying wood in the forest. ***Teleomorph*** not observed. ***Anamorph*** Hyphomycetous. ***Colonies*** effuse, gregarious on host substrate, brown to dark brown. ***Mycelium*** semi-immersed or immersed, pale brown, branched, septate. ***Conidiophores*** absent. ***Conidiogenous cells*** forming directly on creeping hyphae, integrated, monoblastic,determinate. ***Conidia*** 34–61 × 41–63 μm (x̅ = 51 × 51 μm, n = 20), globose to subglobose to turbinate, solitary, olivaceous-green to brown, verrucose and darker at base, with setose appendages on surface. ***Appendages*** with two forms, solitary, cylindrical, unbranched, septate, smooth, brown at base and paler towards to apex, long appendages 51–152 × 3–5 μm (x̅ = 89 × 4.0 μm, n = 20), wide at the base, 2–6-septate, arising from apical part of conidia; short appendages 13–38 × 2.5–6 μm (x̅ = 25 × 4 μm, n = 20), wide at the base, 0–3-septate, arising randomly from conidial apex.

**Figure 2.**
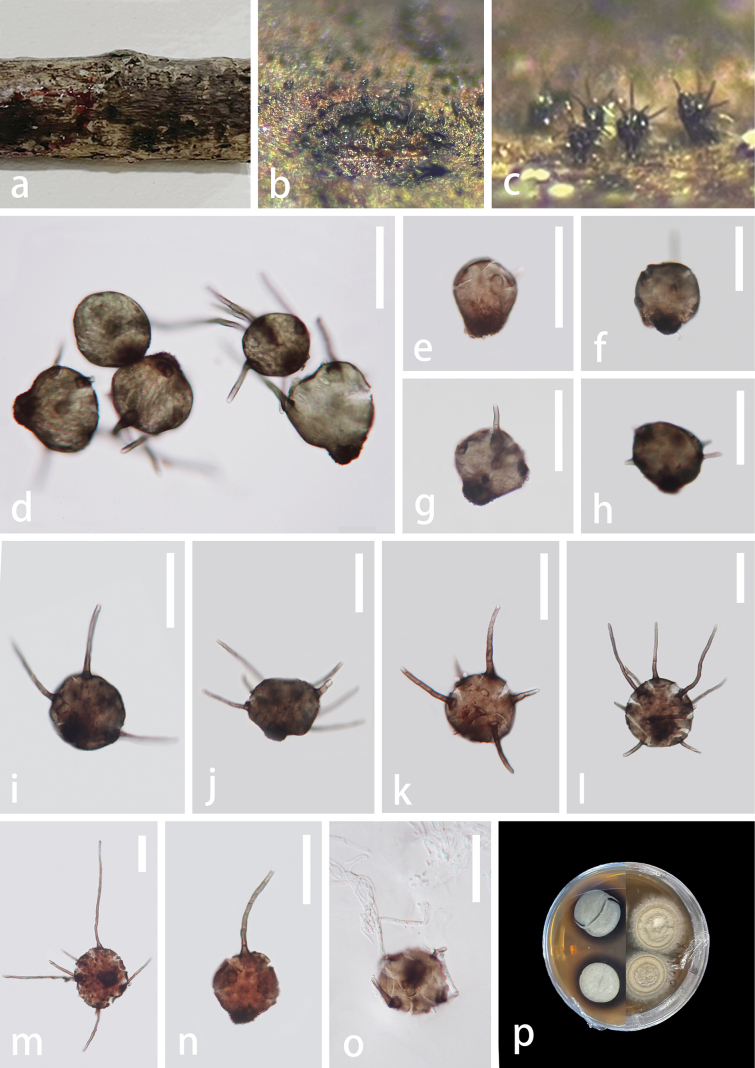
*Polyplosphaeriaguizhouensis* (GZAAS 23–0600, holotype) **a** colonies on decaying wood **b, c** colonies on natural substrates **d–n** conidia bearing appendages **o** germinating conidium **p** colony on PDA (front at right, reverse at left). Scale bars: 50 μm (**d–o**).

#### Culture characteristics.

Conidia germinated on PDA and incubate at room temperature (25 ˚C). Colonies circular, cottony, flat, slightly grey with an undulate margin, forming three concentric zonation, margin regular, brownish grey. The reverse side is greenish grey in the centre, with a dark brown margin and pigment.

#### Material examined.

China, Guizhou Province, Xingyi City, Xianheping National Forest Park, on unidentified decaying wood, 25 September 2021, Xia Tang, xhp08 (GZAAS 23–0600, holotype), ex-type culture GZCC 23–0598.

#### Notes.

The phylogenetic results (Fig. 1) showed that *Polyplosphaeriaguizhouensis* is sister to *Po.pandanicola* within *Polyplosphaeria* with high support (ML = 100, BPP = 1). The comparison of pairwise nucleotides showed that *Polyplosphaeriaguizhouensis* is different from *Po.pandanicola* in 2/801 bp (0.2%) in LSU and 11/460 (2.5%) in ITS. Thus, we describe *Polyplosphaeriaguizhouensis* herein as a novel species in *Polyplosphaeria* following recommendations proposed by Jeewon and Hyde (2016) and Chethana et al. (2021).

### ﻿ Polyplosphaeria hainanensis

Taxon classificationFungiPleosporalesTetraplosphaeriaceae

X. Tang, Jayaward., R. Jeewon & J.C. Kang sp. nov.

4F3ED1E0-39C4-55FE-8E90-0D10F2DC2883

900951

Facesoffungi Number: FoF14665

Figs 3 , 4

#### Etymology.

The specific epithet ‘*hainanensis*’ refers to the place where the fungus was collected, Hainan Province, China.

#### Holotype.

GZAAS 23–0601

#### Description.

***Saprobic*** on unidentified decaying wood in the forest. ***Teleomorph*** not observed. ***Anamorph*** Hyphomycetous. ***Colonies*** effuse, gregarious on host substrate, brown to blackish brown. ***Mycelium*** semi-immersed or immersed, dark brown, branched, septate. ***Conidiophores*** absent. ***Conidiogenous cells*** indistinguishable from creeping hyphae, integrated, monoblastic, determinate. ***Conidia*** 49–134.5 × 52–90.5 μm (x̅ = 86 × 71 μm, n = 20), globose, subglobose, obconical, broadly ellipsoidal to broadly pyriform, variable in shape, sometimes with thin peel on the outer wall of conidia, internally filled with a mass of hyaline, solitary, brown to dark brown, smooth. ***Appendages*** 36–58 × 3–5.5 μm (x̅ = 44.5 × 4 μm, n = 20), cylindrical, solitary, straight or flexuous, unbranched and almost hyaline at the apex, 0–4-septate, smooth, round at apex, pervasive.

**Figure 3.**
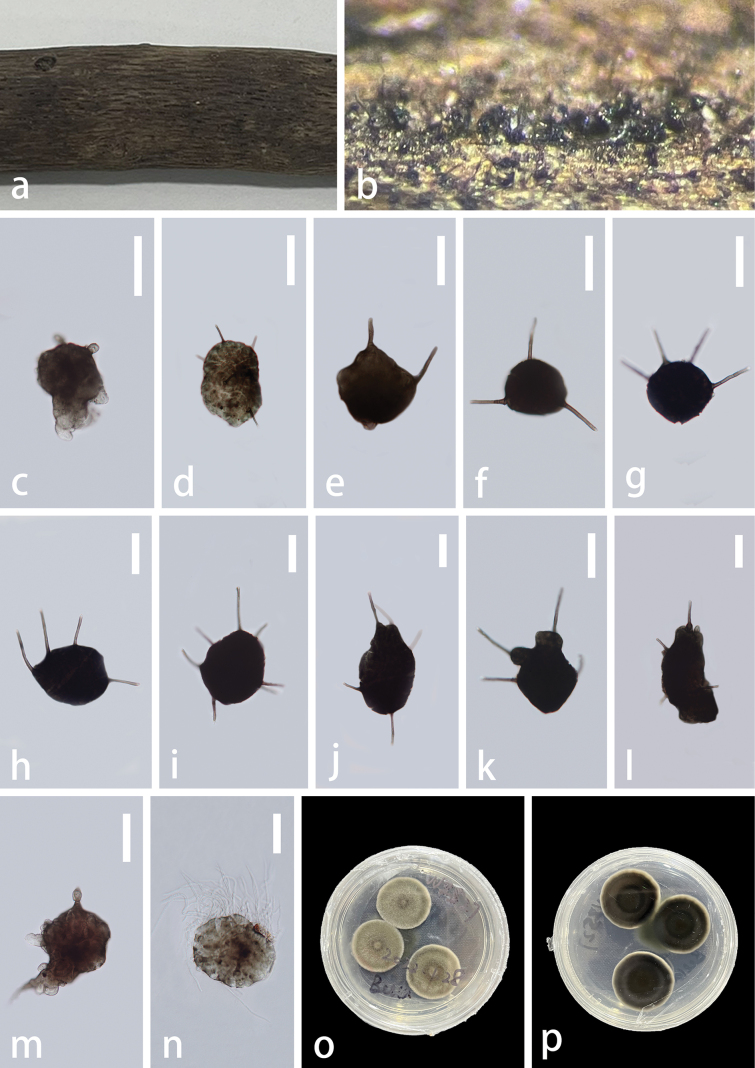
*Polyplosphaeriahainanensis* (GZAAS 23–0601, holotype) **a** colonies on decay wood **b** colonies on natural substrates **c–m** conidia bearing appendages **n** germinating conidium **o** colony on PDA (from front) **p** colony on PDA (from reverse). Scale bars: 50 μm (**c–n**).

#### Culture characteristics.

Conidia germinated from both ends on PDA and incubated at room temperature (25 ˚C). Colonies circular, cottony, flat, olivaceous with a slightly grey entire margin. The reverse side is an olive drab, which gradually extends outwards to form a deep colour ring in the centre with a pale grey margin and no pigment.

**Figure 4.**
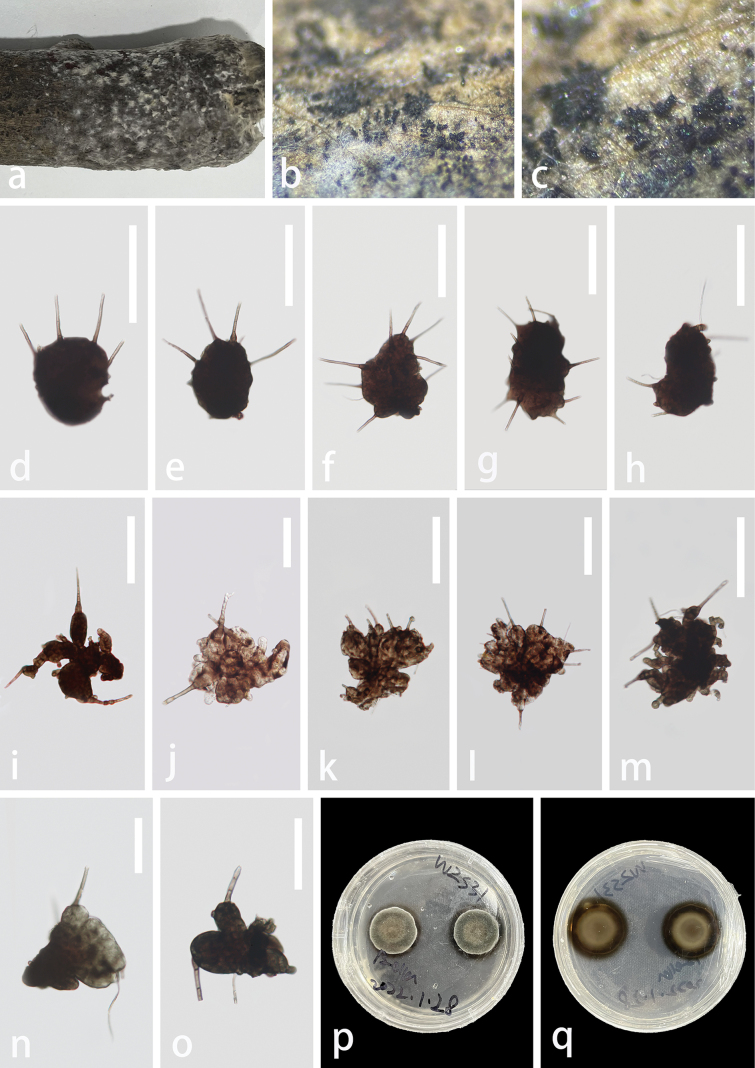
*Polyplosphaeriahainanensis* (GZAAS 23–0602, paratype) **a** colonies on decay wood **b, c** colonies on natural substrates **d–o** conidia bearing appendages **p** colony on PDA (from front) **q** colony on PDA (from reverse). Scale bars: 100 μm (**d–i, k–m**); 50 μm (**j, n, o**).

#### Material examined.

China, Hainan Province, Wuzhishan City, Wuzhishan National Nature Reserve, on unidentified decaying wood, 25 September 2021, Zili Li, WZS27 (GZAAS 23–0601, holotype), ex-type culture GZCC 23–0599; WZS31 (GZAAS 23–0602, paratype), culture GZCC 23–0600.

#### Notes.

Based on the phylogenetic analysis (Fig. 1), two of our *Polyplosphaeria* collections share similar morphology and clustered together with high support (ML = 100, and BPP = 1). The base pair differences between the two strains (GZCC 23–0599 and GZCC 23–0600) were: LSU = 0.2% (2/834), ITS = 0.1% (1/840), respectively, therefore, we considered them as the same species according to the guidelines for species delineation proposed by Jeewon and Hyde (2016). The phylogenetic result (Fig. 1) showed that *Polyplosphaeriahainanensis* is sister to *Po.nabanheensis* within *Polyplosphaeria*. Based on the comparison of the morphological characters with other species in *Polyplosphaeria*, our collection can be distinct in having obconical, broadly ellipsoidal to broadly pyriform, variable conidial shape (without verrucose at the base) and pervasive appendages. The comparison of pairwise nucleotides showed that *Polyplosphaeriahainanensis* is different from *Po.nabanheensis* in 24/826 bp (3%) in LSU, 20/758 (2.6%) in SSU, 17/472 (3.6%) in ITS and 16/344 (5%) in *tub2*. Thus, we describe *Polyplosphaeriahainanensis* herein as a novel species in *Polyplosphaeria* according to the guidelines of Jeewon and Hyde (2016) and Chethana et al. (2021).

### ﻿ Pseudotetraploa

Taxon classificationFungiPleosporalesTetraplosphaeriaceae

Kaz. Tanaka & K. Hirayama, Studies in Mycology 64: 193 (2009)

7B19B5CB-3F31-5C8E-AA3F-71BA3D66C614

515257

Facesoffungi Number: FoF06669

#### Type species.

*Pseudotetraploacurviappendiculata* (Sat. Hatak., Kaz. Tanaka & Y. Harada) Kaz. Tanaka & K. Hirayama, Studies in Mycology 64: 195 (2009).

#### Description.

***Teleomorph morph*** not observed. ***Anamorph Mycelium*** superficial. ***Conidiophores*** absent. ***Conidiogenous cells*** monoblastic, indistinguishable from creeping hyphae. ***Conidia*** composed of 4 to 8 columns, obpyriform to long obpyriform, brown to dark brown, almost smooth, verrucose at the base, pseudoseptate, with setose appendages at the apical part. ***Appendages*** mostly 4, rarely 6 to 8, curved or straight (Tanaka et al. 2009).

#### Notes.

Tanaka et al. (2009) established Pseudotetraploa (Ps.) with three species, which were previously described in *Tetraploa* and typified by *Ps.curviappendiculata* based on a combined SSU and LSU DNA sequence data. *Pseudotetraploa* species are reported as saprobes on bamboo, dead bark of the broad-leaved tree, and unidentified herbaceous plants in Japan, China, and India (Hatakeyama et al. 2005; Tanaka et al. 2009; Hyde et al. 2020a; Yu et al. 2022). *Pseudotetraploa* is only known in its anamorphic state and dwells in terrestrial habitats and contains six species *viz. Ps. bambusicola*, *Ps.curviappendiculata*, *Ps.javanica*, *Ps.longissima*, *Ps.Rajmachiensis*, and *Ps.yunnanensis* (Hatakeyama et al. 2005; Tanaka et al. 2009; Hyde et al. 2020a; Yu et al. 2022; This study; Table 2). In this study, a new *Pseudotetraploa* species isolated from bamboo is introduced.

### ﻿ Pseudotetraploa yunnanensis

Taxon classificationFungiPleosporalesTetraplosphaeriaceae

X. Tang, Jayaward., R. Jeewon & J.C. Kang sp. nov.

320A27B1-7CF0-5B11-BFD0-8238470E1296

900963

Facesoffungi Number: FoF14666

Fig. 5

#### Etymology.

The specific epithet ‘*yunnanensis*’ refers to the place where the fungus was collected, Yunnan Province, China.

#### Holotype.

HKAS 129442.

#### Description.

***Saprobic*** on bamboo. ***Teleomorph*** not observed. ***Anamorph*** Hyphomycetous. ***Colonies*** effuse, gregarious on host substrate, brown to dark brown. ***Mycelium*** superficial, hyaline to pale brown. ***Conidiophores*** absent. ***Conidiogenous cells*** micronematous, mononematous, monoblastic, integrated, usually undistinguishable from superficial hyphae. ***Conidia*** 67–120 × 16.5–35 μm (x̅ = 95 × 24 μm, n = 20), solitary, septate, brown to dark brown, ovoid to obclavate or narrowly obpyriform, consisting of 3–6 columns of cells, rounded at the base 19–36 μm wide (x̅ = 26 μm, n = 20), slightly constricted at septa, rarely branched and make V-shaped conidia; setose appendages at the apical part 15–87 × 3.5–7 μm (x̅ = 37 × 5 μm, n = 20), appendages 3–6 in number, 1–8-septate, brown at the base and almost hyaline at the apex, smooth, unbranched, shorter appendage is straight and longer appendage is curved.

**Figure 5.**
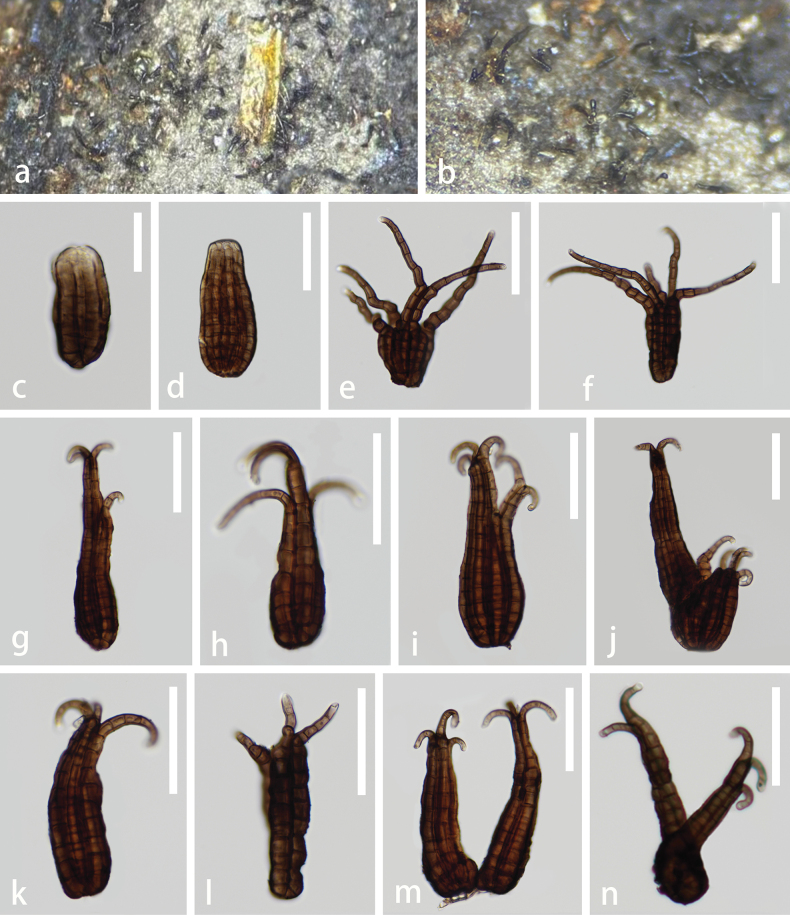
*Pseudotetraploayunnanensis* (HKAS 129442, holotype) **a, b** colonies on natural substrates **c–n** conidia. Scale bars: 20 μm (**c**); 50 μm (**d–n**).

#### Culture characteristics.

Conidia germinated from both ends on PDA and incubated at room temperature (25 ˚C). Colonies circular, cottony, flat, slightly grey with an entire margin, containing a circular white mycelium in the centre. The reverse side is a pale brown in the centre that gradually extends outwards while the colour changes to pale grey, with a brown margin and no pigment.

#### Material examined.

China, Yunnan Province, Puer City, Ailao mountains, on bamboo, May 23, 2022, Rong-Ju Xu, ALS 29 (HKAS 129442, holotype), ex-type culture KUNCC 10464.

#### Notes.

*Pseudotetraploayunnanensis* is similar to *Ps.curviappendiculata* and *Ps.longissima*. However, *Pseudotetraploayunnanensis* differs from *Ps.curviappendiculata* in having branched and V-shaped conidia, consisting of 3–6 columns of cells with 3–6 apical appendages, larger conidia [67–120 μm vs. 52–67(–75) μm] in length and [16–35 μm vs. 15–22 μm] in width, while *Ps.curviappendiculata* consists of 4–5 columns of cells with 4 apical appendages; *Pseudotetraploayunnanensis* differs from *Ps.longissima* in having smaller conidia [67–120 μm vs. (98–)110–148(–155) μm] in length and [16–35 μm vs. 18–25 μm] in width, without verrucose at the base. The phylogenetic analysis showed that *Pseudotetraploayunnanensis* is sister to *Ps.rajmachiensis* and *Ps.javanica*. The comparison of pairwise nucleotides showed that *Pseudotetraploayunnanensis* is different from *Ps.rajmachiensis* in 27/1021 bp (2.6%) in LSU and 30/560 (6%) in ITS; *Pseudotetraploayunnanensis* is different from *Ps.javanica* in 11/1020 bp (1.1%) in LSU and 17/538 (3.2%) in ITS. Thus, we describe *Pseudotetraploayunnanensis* herein as a novel species in *Pseudotetraploa* according to the guidelines Jeewon and Hyde (2016) and Chethana et al. (2021).

### ﻿ Tetraploa

Taxon classificationFungiPleosporalesTetraplosphaeriaceae

Berk. & Broome, Ann. Mag. Nat. Hist. 5: 459, t. 11:6 (1850)

C12C2981-D233-53AB-8DA1-43FC4BF8CDE3

10199

Facesoffungi Number: FoF06666

= Tetraplosphaeria Kaz. Tanaka & K. Hiray., in Tanaka et al., Stud. Mycol. 64: 177 (2009).

#### Type species.

*Tetraploaaristata* Berk. & Broome, Ann. Mag. Nat. Hist. 5: 459 (1850).

#### Description.

***Teleomorph*** see Tanaka et al. (2009). ***Anamorph Tetraploa*** sensu stricto ***Conidiophores*** absent. ***Conidiogenous cells*** monoblastic. ***Conidia*** composed of 4 columns, short-cylindrical, brown, verrucose at the base, euseptate, with 4 setose appendages at the apex (Tanaka et al. 2009).

#### Notes.

Tanaka et al. (2009) established *Tetraplosphaeria* to accommodate pleosporalean species that have massarina/lophiostoma-like teleomorph and anamorphs belonging to *Tetraploa* sensu stricto based on a combined SSU and LSU DNA sequence data. Later, Hyde et al. (2013) treated *Tetraploa* as a synonym of *Tetraplosphaeria*, which has been applied previously to anamorphic species and used *Tetraploa* instead of *Tetraplosphaeria*. Species of *Tetraploa* are mainly reported as saprobes, distributed in freshwater and terrestrial habitats, and only *T.aristata* has been reported as a pathogen on various plants and human pathogen that cause cysts (Markham et al. 1990; Tanaka et al. 2009; Hyde et al. 2013; Liao et al. 2022). *Tetraploa* has been recovered from more than 80 plants, such as bamboo culms, submerged wood, palms, and *Poaceae*, on the leaves of Acer and liverworts (Ellis 1949; Ando 1992; Hyde et al. 2013; Liao et al. 2022). Saxena et al. (2021) mentioned that *Frasnacritetrus* is probably a fossil of *Tetraploa*. Nuñez Otaño et al. (2022) considered *Frasnacritetrus* as a synonym of *Tetraploa* and transferred five *Frasnacritetrus* fossil species into *Tetraploa viz. Tetraploaconata*, *T.indica*, *T.josettae*, *T.siwalika* and *T.taugourdeaui* based on the observation that the spores of both fossil and contemporary species exhibit identical morphological characteristics. To date, there are 35 species accepted in *Tetraploa* (Wijayawardene et al. 2022; Jayawardena et al. 2023; this study; Table 2). In this study, a new *Tetraploa* species is introduced.

### ﻿ Tetraploa hainanensis

Taxon classificationFungiPleosporalesTetraplosphaeriaceae

X. Tang, Jayaward., R. Jeewon & J.C. Kang sp. nov.

5D3715B1-81F9-51B1-9727-C1CA53002B62

900952

Facesoffungi Number: FoF14667

Figs 6 , 7

#### Etymology.

The specific epithet ‘*hainanensis*’ refers to the place where the fungus was collected, Hainan Province, China.

#### Holotype.

GZAAS 23–0603.

#### Description.

***Saprobic*** on unidentified decaying wood in forest. ***Teleomorph morph*** Not observed. ***Anamorph*** Hyphomycetous. ***Colonies*** effuse, gregarious on host substrate, brown to dark brown. ***Mycelium*** semi-immersed or immersed, pale brown, branched, septate. ***Conidiophores*** absent. ***Conidiogenous cells*** integrated, monoblastic, determinate. ***Conidia*** 30–46 × 18–36 μm (x̅ = 38 × 27 μm, n = 20), cylindrical with obtuse ends, pale brown to brown, verrucose, composed of four columns of cells, sometimes five columns of cells, 4–5-septate in each column, smooth, mostly with four apical appendages, some with one or two or five appendages. ***Appendages*** 52–209 × 3–6 μm (x̅ = 140 × 4 μm, n = 20) cylindrical, solitary, unbranched, guttulate, septate, wide at the base, divergent, pale brown to brown, 5–16-septate, straight or slightly flexuous, smooth-walled.

**Figure 6.**
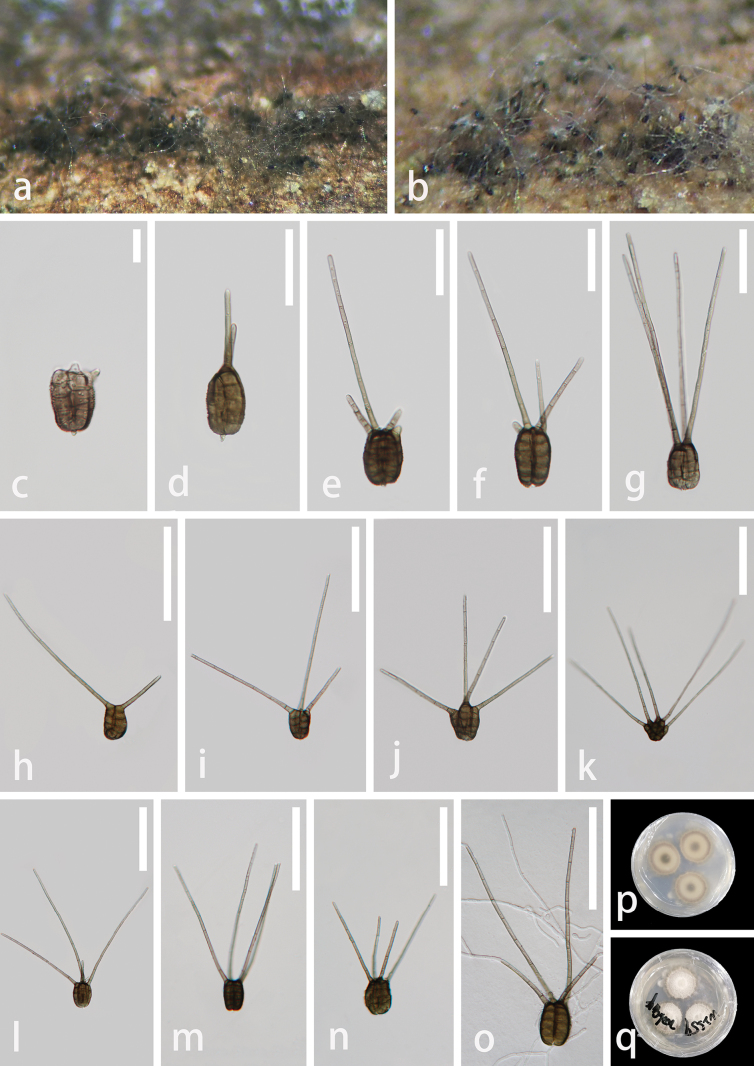
*Tetraploahainanensis* (GZAAS 23–0603, holotype) **a, b** colonies on natural substrates **c–n** conidia bearing 1–5 appendages **o** germinating conidium **p** colony on PDA (from reverse) **q** colony on PDA (from front). Scale bars 20 μm (**c**); 50 μm (**d–g**); 100 μm (**h–o**).

#### Culture characteristics.

Conidia germinated from both ends on PDA and incubated at room temperature (25 ˚C). Colonies circular, cottony, flat, slightly grey with an entire margin, contain a circular white mycelium in the centre. The reverse side is a pale brown in the centre that gradually extends outwards while the colour changes to pale grey, with a brown margin and no pigment.

**Figure 7.**
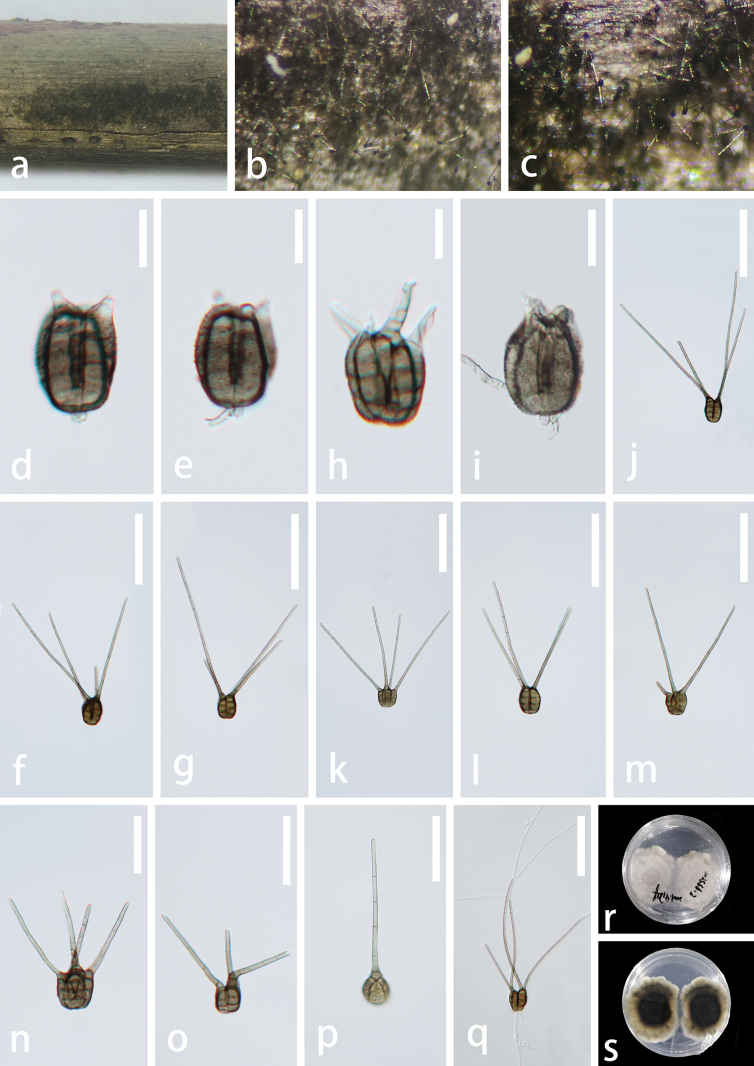
*Tetraploahainanensis* (GZAAS 23–0604, paratype) **a** colonies on decay wood **b, c** colonies on natural substrates **d–p** conidia bearing 1–4 appendages **q** germinating conidium **r** colony on PDA (from front) **s** colony on PDA (from reverse). Scale bars: 20 μm (**d–g**); 100 μm **(h–l, o, q**); 50 μm (**m, n, p**).

#### Material examined.

China, Hainan Province, Wuzhishan City, Wuzhishan National Nature Reserve, on unidentified decaying wood, 25 September 2021, Zili Li, WZS59 (GZAAS 23–0603, holotype), ex-type culture GZCC 23–0601; WZS66.2 (GZAAS 23–0604, paratype), culture GZCC 23–0602.

#### Notes.

*Tetraploahainanensis* is morphologically similar to *T.pseudoaristata*. However, *Tetraploahainanensis* can be distinguished from *T.pseudoaristata* in having larger conidia (30.5–46 × 18–36 μm vs. 22–31 × 15–20 µm) with four columns of cells, sometimes five columns of cells, and longer appendages (52–209 × 3–6 μm vs. 23–107 × 2–5 μm), commonly four in number, sometimes five. Based on the phylogenetic analysis, two of our *Tetraploa* collections which share similar morphology clustered together with high support (ML = 100, and BPP = 1 (Fig. 1)). The base pair differences between the two strains were: LSU = 0.1% (1/806), ITS = 0% (0/516), and *tub2* = 0% (1/633), respectively. Therefore, we considered them as the same species according to the guidelines for species delineation proposed by Jeewon and Hyde (2016). *Tetraploahainanensis* forms a distinct lineage but close to *T.yakushimensis* and *T.tetraploa*. However, *Tetraploahainanensis* differs from *T.yakushimensis* by having four or five columns and appendages, while *T.yakushimensis* has only four columns and appendages; *Tetraploahainanensis* differs from *T.tetraploa* in having four or five columns and shorter appendages (52–209 × 3–6 μm vs. 263–350 × 2–3 μm), while *T.tetraploa* has only four columns and slender appendages. The comparison of pairwise nucleotide showed that *Tetraploahainanensis* is different from *T.yakushimensis* in 31/620 bp (3%) in LSU, 7/814 (0.98%) in ITS, and 87/450 (19%) in *tub2* and *Tetraploahainanensis* is different from *T.tetraploa* in 31/620 bp (3%) in LSU, 7/814 (0.98%) in ITS, and 87/450 (19%) in *tub2*. Based on the combination of morphological characters and multigene phylogeny, we describe *Tetraploahainanensis* herein as a distinct species according to the guidelines of Jeewon and Hyde (2016) and Chethana et al. (2021).

## ﻿Discussion

Hyde et al. (2018) reported that more than 95% of fungi collected in northern Thailand could be new to science and there is a dire need to collect more samples from a wide variety of hosts to better understand fungal diversity estimates. In the same way, fungal diversity in Yunnan, Guizhou and Hainan is expected to be rather high. In this study, collections of decayed wood samples and bamboo were done to assess which fungal species are potentially colonising them. Our study reveals four anamorphic fungal species that belong to the family Tetraplosphaeriaceae. In this study, we characterise two new anamorphic species collected from unidentified decayed wood samples that belong to *Polyplosphaeria*. In *Polyplosphaeria* this study brings the number of species to six. The first new species is described as *Po.guizhouensis* and our multigene phylogeny depict a close relationship to *Po.pandanicola*. The latter was collected from fallen dead and decaying leaves of *Pandanus* sp. in China (Tibpromma et al. 2018) and characterized by micronematous conidiophores; monoblastic, incomplete globose connected to base of conidia conidiogenous cell with guttules, hyaline; globose to subglobose, solitary, verrucose at base conidia with almost hyaline at apex, unbranched setose appendages on surface. However, *Po.guizhouensis* differs in having turbinate conidia which are verrucose and darker at the base. Furthermore, it possesses a longer conidium base and two types of appendages originating from the apical part of the conidia. With regard to DNA sequence data comparison, *Po.guizhouensis* differs from *Po.pandanicola* (MFLUCC 17–2266) in having 11 out of 460 (2.5%) and 2 out of 801 (0.2%) different base pairs (bp) in the ITS alignments and LSU gene respectively. Our second new species, named *Polyplosphaeriahainanensis*, forms a strongly supported subclade with *Po.nabanheensis*. The latter was collected from fallen dead and decaying leaves of *Pandanus* sp. in China (Tibpromma et al. 2018). It is characterised by monoblastic, hyaline conidiogenous cells with guttules; oval to ellipsoid conidia, made up of 2–3 cells, and verrucose at base, rough-walled, with apical setose appendages. However, *Po.hainanensis* differs with regards to having an obconical, broadly ellipsoidal to broadly pyriform, variable shaped conidia (no verrucose at the base) and pervasive appendages. Comparison of available LSU, ITS, SSU and *tub2* sequences also reveal differences in base pairs that support species distinctiveness. For instance, *Po.hainanensis* differs from *Po.nabanheensis* in having 24/826 bp (3% difference) in LSU, 20/758 (2.6% difference) in SSU, 17/472 (3.6% difference) in ITS and 16/344 (5% difference) in *tub2*. Another peculiar finding when we analysed the relationships of *Polyplosphaeria* species, we found that two strains of *Polyplosphaeriathailandica* (MFLU 15–3273) and *Aquatisphaeriathailandica* (MFLUCC 21–0025 and DLUCC B151) clustered together with 75% ML/0.99 BPP support and sister to species of *Shrungabeeja*. However, it’s important to note that this relationship lacked significant statistical support, a pattern observed in various previous studies as well (Li et al. 2021; Liao et al. 2022). *Aquatisphaeriathailandica* has been reported as a saprobe on submerged decaying wood in China. It is characterised by macronematous, mononematous, solitary, unbranched conidiophores with 3–4 septa; monoblastic, integrated, terminal, subcylindrical conidiogenous cells; and acrogenous, solitary, subglobose or turbinate, muriform, dictyoseptate conidia with 3–4 (mostly 4) cylindrical, upward appendages with 1–2-septa. At the same time, *Po.thailandica* is recognised as a saprobe found in decaying bamboo in Thailand. It is characterised by monoblastic conidiogenous cells; acrogenous, solitary, globose, obovoid, pyriform, ellipsoidal, obconical, muriform, verrucose conidia with 2–5-septate appendages, occasionally, two conidia are associated together at the basal cell (Li et al. 2016; Li et al. 2020). Based on the phylogenetic analyses, it seems that *Po.thailandica* is a member of *Aquatisphaeria*. There is not much taxonomic data available for *Aquatisphaeria*, hence we recommend that further collections of this genus are required to elucidate its relationships to *Po.thailandica*. Alternatively, there might be a need to relook into the taxonomy of *Po.thailandica* and verify whether the DNA sequences submitted are reliable.

The third anamorphic species was collected from bamboo in Yunnan, China and subsequently assigned to *Pseudotetraploa*. To date, five species have been reported, and this study extends the known species count to six. The new species is described as *Ps.yunnanensis* and our multigene phylogeny depict a close relationship to *Ps.rajmachiensis*. The latter was collected from decaying bamboo culms in India (Hyde et al. 2020a) and characterised by the absence of conidiophores; micronematous, mononematous, monoblastic conidiogenous cells; ovoid to obclavate or obpyriform conidia, minutely verrucose at the base; unbranched, septate, setose appendages at the apical part, consisting of two appendages with one straight and one curved. However, *Ps.yunnanensis* differs in having larger conidia that rarely separate, consisting of 3–6 columns of cells, forming a V-shape conidia, 3–6 apical appendages. In terms of DNA sequence data comparison, *Ps.yunnanensis* differs from *Ps.rajmachiensis* (NFCCI 4618) in LSU by 27/1021 bp (2.6% difference) and in ITS by 30/560 bp (6% difference).

The last anamorphic species was collected from unidentified decaying wood in Hainan, China, and was assigned to *Tetraploa*. With the addition of this species, the genus now comprises a total of 35 species. The new species is described as *T.hainanensis*, and the multigene phylogeny depicts a close relationship to *T.yakushimensis*. The latter was collected on culms of *Arundodonax* in Japan (Tanaka et al. 2009) and characterised by the absence of conidiophores; monoblastic conidiogenous cells; solitary, short cylindrical, brown, verruculose conidia, composed of 4 columns with 4 apical setose appendages. However, *T.hainanensis* differs in having four or five culms and appendages. In terms of DNA sequence data comparison, *T.hainanensis* differs from *T.yakushimensis* (KT 1906) in 31/620 bp (3% difference) in LSU, 7/814 (0.98% difference) in ITS, and 87/450 (19% difference) in *tub2*.

Tetraplosphaeriaceae is a well-known family in terrestrial habitats with mostly saprobes being reported so far, and previous and recent studies have shown that Tetraplosphaeriaceae is widely associated with many plants in different countries. In this work, we describe four new Tetraplosphaeriaceae species based on phylogenetic and morphological comparisons with allied taxa, update the phylogeny of the Tetraplosphaeriaceae family and also provide a checklist of species with other details (Table 2). To date, there are 69 species in Tetraplosphaeriaceae, of which 23 species (including this study) are from China. This study enriches the diversity of fungi in China of Tetraplosphaeriaceae species.

## Supplementary Material

XML Treatment for Tetraplosphaeriaceae

XML Treatment for Polyplosphaeria

XML Treatment for Polyplosphaeria guizhouensis

XML Treatment for Polyplosphaeria hainanensis

XML Treatment for Pseudotetraploa

XML Treatment for Pseudotetraploa yunnanensis

XML Treatment for Tetraploa

XML Treatment for Tetraploa hainanensis

## ﻿Additional information

### Author ORCIDs

Xia Tang https://orcid.org/0000-0003-2705-604X

Rajesh Jeewon https://orcid.org/0000-0002-8563-957X

Yong-Zhong Lu https://orcid.org/0000-0002-1033-5782

Abdulwahed Fahad Alrefaei https://orcid.org/0000-0002-3761-6656

Ruvishika S. Jayawardena https://orcid.org/0000-0001-7702-4885

Rong-Ju Xu https://orcid.org/0000-0002-3968-8442

Jian Ma https://orcid.org/0009-0008-1291-640X

Xue-Mei Chen https://orcid.org/0009-0004-8631-0735

Ji-Chuan Kang https://orcid.org/0000-0002-6294-5793

### Data availability

All of the data that support the findings of this study are available in the main text. DNA sequences generated have been submitted to Genbank.
